# Involvement of 27-Hydroxycholesterol in Mitotane Action on Adrenocortical Carcinoma

**DOI:** 10.3390/cells9040885

**Published:** 2020-04-04

**Authors:** Antonina Germano, Daniela Rossin, Valerio Leoni, Noemi Iaia, Laura Saba, Vittoria Basile, Soraya Puglisi, Claudio Caccia, Giuseppe Poli, Fiorella Biasi, Massimo Terzolo

**Affiliations:** 1 Department of Clinical and Biological Sciences, University of Turin, 10043 Orbassano (Turin), Italy; d.rossin@unito.it (D.R.); noemi.iaia@unito.it (N.I.); laura.saba@unito.it (L.S.); basile_vittoria@libero.it (V.B.); soraya.puglisi@unito.it (S.P.); giuseppe.poli@unito.it (G.P.); fiorella.biasi@unito.it (F.B.); 2 Department of Oncology, University of Turin, 10060 Candiolo (Turin), Italy; antonina.germano@unito.it; 3 Department of Medicine and Surgery, University of Milano-Bicocca, Laboratory of Clinical Pathology, 20832 Desio (Milan), Italy; valerio.leoni@unimib.it; 4 Unit of Medical Genetics and Neurogenetics, Fondazione IRCCS Istituto Neurologico Carlo Besta, 20131 Milan, Italy; Claudio.Caccia@istituto-besta.it

**Keywords:** mitotane, adrenocortical carcinoma, cholesterol metabolism, 27-hydroxycholesterol, oxysterols, cytotoxicity, mitochondria, caspase-3, apoptosis

## Abstract

Adrenocortical carcinoma (ACC) is a rare cancer with poor prognosis. Mitotane, the standard treatment for ACC, impairs adrenocortical steroid biosynthesis and cholesterol metabolism. In the H295R cell line, a standard ACC in vitro model, mitotane was previously reported to enhance the production of some oxysterols. To verify the possible mechanistic involvement of oxysterols in the anti-ACC effect of mitotane, a gas chromatography mass spectrometry (GC-MS) profiling of oxysterols and the main cholesterol precursors was carried out in H295R cells. Among the oxysterols detected in mitotane-treated cells, 27OHC was markedly produced, as well as lanosterol and lathosterol cholesterol precursors. In this cell model, mitotane was confirmed to affect mitochondrial transmembrane potential and induce apoptosis. Such cytotoxic effects were perfectly matched by H295R cell treatment with a single identical micromolar amount of 27OHC. The mitotane-dependent strong increase in 27OHC was confirmed in vivo, in the plasma of ACC patients under treatment with the drug. Moreover, lanosterol, lathosterol, desmosterol and, to a minor extent, 24-hydroxycholesterol and 25-hydroxycholesterol plasma levels were significantly increased in those patients. The cytotoxic effect of mitotane on ACC cells may be partly related to the increased intracellular level of 27OHC induced by the drug itself.

## 1. Introduction

Adrenocortical carcinoma (ACC) is a rare, aggressive endocrine tumor usually associated with hypercortisolism and characterized by a poor prognosis [1]. The incidence is about two cases per million per year, with an overall five-year survival rate ranging between 16% and 47%, which is mainly influenced by tumor stage [2,3]. The only approved treatment of ACC relies on an old adrenolytic drug, mitotane, a derivate of the insecticide dichlorodiphenyltrichloroethane (DDT). In clinical practice, mitotane has shown to be effective both in adjuvant settings and in the treatment of advanced/progressive diseases [4,5], but is associated with a number of adverse events, including hypoadrenalism, hypercholesterolemia, hypertriglyceridemia and central hypothyroidism [6].

Mitotane acts as a steroidogenesis inhibitor inducing mitochondrial functional impairment and adrenal cortex necrosis, even if the precise mechanism of action is far from being elucidated. Moreover, it was reported that an increase in mevalonic acid during mitotane administration was associated with increased cholesterol synthesis [7]. These authors have shown that this effect is due to the known drug ability to block CYP450-mediated reactions, thus influencing formation of cholesterol oxidized metabolites responsible for down-regulating hepatic cholesterol synthesis.

Many studies have shown that the mevalonate pathway is up-regulated in several cancers such as leukemia, lymphoma, multiple myeloma and endocrine tumors [8,9,10]. Furthermore, cholesterol depleting agents were used to treat different types of cancers in preclinical models [11,12]. However, the mechanistic involvement of cholesterol precursors and/or metabolites in ACC progression and in the therapeutic activity of mitotane are, so far, almost unexplored. Only a single—while seminal—report is available showing that mitotane is able to induce specific changes in lipid metabolism in ACC cells [13]. In that in vitro study, employing the ACC reference cell line H295, relatively high concentrations of mitotane were reported to overturn cholesterol synthesis and lead cells to apoptosis, possibly through the induction of endoplasmic reticulum (ER) stress. Mitotane was demonstrated to markedly increase the steady-state level of cholesterol precursors lanosterol and lathosterol in H295 ACC cells, but also a couple of oxysterols of non-enzymatic origin, namely 7-ketocholesterol and 7β-hydroxycholesterol [13], the latter two compounds being well known as potentially toxic lipid oxidation products [14,15].

Based on those findings, the main aim of this study was to provide additional mechanistic hints on both the beneficial and adverse effects of mitotane, by focusing on the oxysterol profile induced by this drug in the H295 cell line and also directly in ACC patients. Specific oxysterols might be relevant for the action of mitotane at the cellular level and could represent useful response markers to be exploited in clinical practice.

Here, we report on: (1) the specific increase of 27OHC, the major oxysterol of pathophysiological relevance, in H295R cells challenged with a mitotane concentration confirmed to exert toxic and proapoptotic effects; (2) the reproduction of such observed mitotane toxicity in vitro by adding an identical micromolar amount of 27OHC instead of the drug to the incubation medium of H295R cells; (3) the marked increase of 27OHC in the peripheral blood of ACC patients, under therapy with mitotane.

## 2. Materials and Methods

### 2.1. Cell Culture and Chemical Reagents

The H295R cell line, the adherent variant of H295 cell line, was supplied from the American Type Culture Collection (ATCC, Rockville, MD, USA). The H295R cells were cultured in a 1:1 mixture of Dulbecco’s Modified Eagle’s Medium (DMEM) (Corning, New York, NY, USA) and Ham’s F-12 Nutrient mixture (F12) (Corning, New York, NY, USA) supplemented with 2 mmol/L L-glutamine, penicillin (25 units/mL), and streptomycin (25 mg/mL, all from Sigma-Aldrich, Saint Louis, MO, USA) and 2.5% of Nu-Serum (Corning, New York, NY, USA) and enriched with 1% di ITS + Premix (Corning). Cells were incubated with mitotane (Supelco, Bellefonte, USA) dissolved in 100% methanol (Sigma-Aldrich, Saint Louis, MO, USA) or 27OHC (Avanti Polar Lipids, Alabaster, AL, USA). dissolved in 100% ethanol.

All oxysterols of enzymatic origin detectable in human plasma [16] were considered, namely 24-hydroxycholesterol, 25-hydroxycholesterol, 27-hydroxycholesterol, 7-α-hydroxycholesterol, plus the two oxysterols of non-enzymatic origin, namely 7-ketocholesterol and 7-β-hydroxycholesterol, already detected in the mitotane-treated H295 cell line [13]. Their deuterated forms used as internal standards for gas chromatography mass spectrometry analyses were obtained from Avanti Polar Lipids (Alabaster, AL, USA). All other not specified reagents and chemicals were obtained from Sigma Aldrich (Saint Louis, MO, USA).

### 2.2. Recruited Individuals and Patients

For the reported study, we selected 14 ACC patients and 5 healthy individuals. Their plasma samples were part of a large biorepository of biological samples available at the University of Torino, Internal Medicine-San Luigi Hospital (Orbassano, Turin, Italy). The study protocol includes storage of biomaterials of ACC patients with the aim to search for predictors of response to treatment and prognostic markers. In agreement with the ethical guidelines of the 1975 Declaration of Helsinki, it was approved by the local Ethical Committee (San Luigi Hospital, Orbassano, Italy, n. 36/2012). All the selected plasma samples were anonymized by an Internal Medicine staff member not involved in the study. Inclusion criteria for ACC patients were as follows: pathologically confirmed diagnosis of ACC, mitotane therapy for at least 12 months, regular determination of plasma mitotane concentration, mitotane plasma concentration in therapeutic range (14–20 mg/L), total cholesterol levels >190 mg/dL after mitotane treatment (Table 1). Tumor stage was established according to the ENSAT classification: stage I, confined tumor, ≤5 cm; stage II, confined tumor, >5 cm [3]. Clinical data were available for all patients. A written informed consent for the analysis of plasma oxysterols and related intermediate products of cholesterol was obtained from each person recruited in the study (Table 1).

### 2.3. Cholesterol Precursors and Oxysterols Quantification

For in vitro quantification, H295R cells were seeded into 6-well plates and treated with mitotane (5 or 10 µM) for 24, 48 or 72 h, as described in the result section. After the incubation times, ACC cell pellets were collected then added to a screw-capped vial sealed with a Teflon septum together with 1000 ng of D7-lathosterol, 50 ng of D6-lanosterol, 50 ng of D7-7α-hydroxycholesterol, 50 ng of D7-7β-hydroxycholesterol, 50 ng of D7-7-oxo-cholesterol, 50 ng of D6-24-hydroxycholesterol, 50 ng of D6-25-hydroxycholesterol, 50 ng of D6-27-hydroxycholesterol as internal standards, plus 50 μL of butylated hydroxytoluene (5 g/L) and 50 μL of K3- ethylenediaminetetraacetic acid (EDTA) (10 g/L) to prevent auto-oxidation. Each vial was flushed with argon for 10 min to remove air. Alkaline hydrolysis was allowed to proceed at room temperature (22 °C) with magnetic stirring for 60 min in the presence of ethanolic 1 M potassium hydroxide solution. After hydrolysis, the sterols were extracted twice with 5 mL cyclohexane. Oxysterols were eluted on solid phase extraction (SPE) cartridge (Waters, Milford, MA, USA) by isopropanol:hexane 30:70 v/v. The organic solvents were evaporated under a gentle stream of argon and converted into trimethylsilyl ethers with N,O-Bis(trimethylsilyl)trifluoroacetamide (BSTFA).

For in vivo quantification, 0.25 mL of plasma of ACC patients and control were added to a screw-capped vial sealed with a Teflon septum, together with deuterium labelled or structural homologous internal standards, butylated hydroxytoluene (BHT) and EDTA and processed as described above.

Gas chromatography mass spectrometry (GC-MS) analysis was performed on a GC equipped with an Elite column (30 m × 0.32 mmid × 0.25 mm film; Perkin Elmer, Waltham, MA, USA) and injection was performed in splitless mode and using helium (1 mL/min) as a carrier gas. The temperature program was as follows: initial temperature of 180 °C was held for 1 min, followed by a linear ramp of 20 °C/min to 270 °C, and then a linear ramp of 5 °C/min to 290 °C, which was held for 10 min. The mass spectrometer operates in the selected ion-monitoring mode. Peak integration was performed manually, and sterols were quantified from selected-ion monitoring analysis against internal standards using standard curves for the listed sterols. Additional qualifier (characteristic fragment ions) ions were used for structural identification [17,18,19].

### 2.4. Oxysterol Challenge and Cytotoxicity Assay

H295R cells were seeded into 96-well plates in triplicates and incubated for 48 h in the absence or in the presence of increasing concentrations of 27-hydroxycholesterol or mitotane (2.5, 5, 7.5, 10 µM) for 48 h. Concentrations of 27OHC are in a range comparable with the oxysterol’s levels quantified in ACC cell supernatants by GC-MS analysis. The cell proliferation reagent WST-1 (Roche Applied Science, Penzberg, Germany), based on recording formazan dye production by metabolically intact cells, was used to measure the potential toxic effect of different concentrations of 27OHC and mitotane on H295R cells. The tetrazolium salt WST-1 was added to each well at the end of cell treatments with mitotane or 27OHC: the produced water-soluble formazan was measured after 30 min by using a microplate reader (iMARK microplate reader, Biorad Life Science Group, Hercules, CA USA) at 450 nm wavelength (630 nm was considered as reference wavelength).

### 2.5. JC-1 Labelling

H295R cells were incubated with 5, 10 µM mitotane or 5, 10 µM 27-hydroxycholesterol at 37 °C for 48 h. Cells were pre-incubated with 10 µg/mL JC-1 dissolved in DMEM/F12 at 37 °C for 10 min. The cationic dye 5,5′,6,6′-Tetrachloro1,1′,3,3′-tetraethyl benzimidazolylcarbocyanine iodide (JC-1) (Life Technologies, Monza, Italy) exhibits membrane-potential-dependent accumulation in mitochondria as J-aggregates that can be detected by red fluorescence. When mitochondria are depolarized, JC-1 is converted into a monomer displaying green fluorescence, which is considered as an index of apoptosis; the red fluorescence (590 nm) and green fluorescence (530 nm) were detected by using a 96-well plate Luminometer (GloMax, Promega, Milan, Italy). Mitochondrial polarization state was evaluated as the ratio between red and green fluorescence.

### 2.6. Evaluation of Caspase-3 Activity

After 48 h treatment with mitotane, H295R cells were collected by centrifugation, washed with cold 0.1 M phosphate buffered saline (PBS), and suspended in 600 μL ice-cold lysis buffer [25 mM 4-2-hydroxyethyl-1-piperazineethansulphonic acid (HEPES), 5 mM MgCl2, 5 mM EDTA, 5 mM dithiothreitol, 2 mM phenylmethylsulphonyl fluoride] for 15 min; the suspension was frozen/thawed 10 times (liquid nitrogen/37 °C) and centrifuged at high speed for 30 min at 4 °C and the supernatant was collected. The samples were diluted to achieve a protein concentration of 0.5 mg/mL and incubated for 45 min at 37 °C with 65 μM (final concentration) fluorogenic substrate N-Acetyl-Asp-Glu-Val-Asp-7-amino-4-methylcoumarin (Ac-DEVD-AMC). Caspase-3 activity was evaluated as the release of fluorescent 7-amino-4-methylcoumarin (AMC), using a 96 Microplate Luminometer (GloMax, Promega, Milan, Italy) and recording Δ min fluorescence at 380 nm excitation and 510 nm emission. Fluorescence was referred to AMC standard curve (ranging from 10 nM to 10 µM). Proteins were evaluated with the Bio-Rad protein assay dye reagent (Bio-Rad, Milano, Italy).

### 2.7. Statistical Analysis

Statistical differences between mitotane treated and untreated cells and between ACC patients and controls were evaluated using Student’s t-test and a one-way Analysis of Variance (ANOVA) test associated with the Bonferroni’s multiple comparison post-test. Data were analyzed with GraphPad Prism 6 software (San Diego, CA, USA) and results were expressed as mean ± Standard Deviation (SD).

## 3. Results

### 3.1. Effect of Mitotane on Cholesterol Precursors and Oxysterols Profile in the Standard Human Adrenal Cancer Cell Line

A net modification in the pattern of cholesterol precursors and oxysterols was observed in H295R cells after a 48 h exposure to 10 µM mitotane. As reported in Table 2, total cholesterol recovered by GC-MS in mitotane treated H295R cells showed a 1.5-times increase as compared to untreated cells. In addition, lanosterol and lathosterol resulted significantly higher in the mitotane-treated cell pellets than in untreated cell pellets, showing a 1.7- and 1.6-times increase respectively; conversely, desmosterol markedly dropped in mitotane treated H295R cells, being five times lower than in control cells. Again, in Table 2, the content of 7KC and 7βOHC did not show any significant difference between treated and untreated cells. Moreover, a slight significant decrease was observed for 7αOHC, the only B ring enzymatic oxysterol of pathophysiological relevance [14,15]. With regard to the side chain oxysterol of major relevance detectable in human peripheral blood, 24OHC and 25OHC were practically undetectable in H295R cells both mitotane-treated or not. Interestingly, only 27OHC was detectable in this cell line, and the adopted 10 µM mitotane treatment stimulated its cellular content up to 4.6 times as to the constitutive level (Table 2).

**Figure 1:**
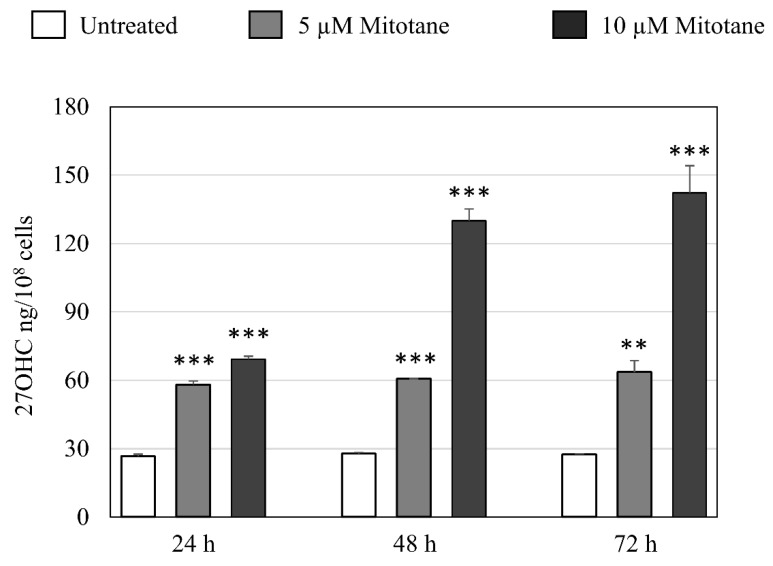
Production of 27-Hydroxycholesterol by H295R cell treatment with mitotane. H295R cells were incubated at 37 °C up to 72 h, in the absence or in the presence of two different concentrations of mitotane, namely 5 and 10 µM. Data are reported as means ± SD from three independent experiments, after normalization for the cell number, and are expressed in ng/10^8^ cells. Significantly different versus untreated cells: ** *p*< 0.01; *** *p* < 0.001.

Further assessment of 27OHC intracellular level against time was performed, also including a 5-µM mitotane treatment. As reported in Figure 1, in the presence of either 5- or 10-µM mitotane a net increase of the H295R oxysterol content was observed already after 24 h incubation. While the 27OHC content remained stable within 5-µM mitotane-treated cells, it kept increasing with time in 10-µM mitotane-treated cells.

### 3.2. Effect of Mitotane and 27OHC on Viability of H295R Human Adrenal Cancer Cells

**Figure 2:**
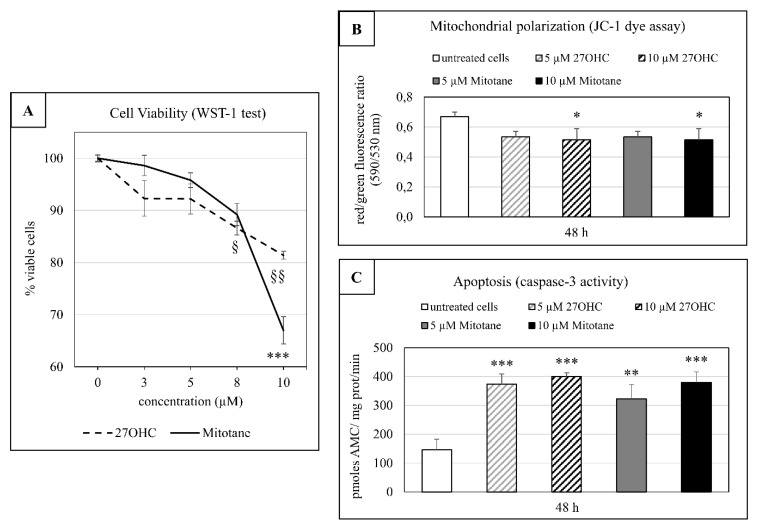
Effect of 27-hydroxycholesterol and mitotane on H295R cell viability and apoptosis. (**A**) Cells were treated for 48 h with increasing concentrations of 27OHC or mitotane (0–10 µM). For the WST-1 test see Methods 2.4. Data are reported as means ± SD from three independent experiments and expressed as % of viable cells compared to control (untreated cells): ^§^ *p* < 0.05, ^§§^ *p* < 0.01, *** *p* < 0.001. (**B**) Mitochondrial polarization state in H295R cells treated with 5 or 10 µM 27OHC or with 5 or 10 µM mitotane for 48h. For the JC-1 assay see Methods 2.5. Data are means ± S.D. of three different experiments. Significantly different versus untreated cells: * *p* < 0.05. (**C**) Caspase-3 specific activity in H295R cells treated with 5 or 10 µM 27OHC or mitotane for 48h. AMC: 7-amino-4-methylcoumarin (see Methods 2.6.). Data are means ± S.D. of three different experiments. Significantly different versus untreated cells: ** *p* < 0.01, *** *p* < 0.001.

Here, preliminary unreported analyses showed that the 48-h challenge with 27OHC actually affected H295R cell viability exhibiting an IC50 of 28.72 µM ± 1.87. On this basis, all further experimental observations were carried out at 48 h of incubation time, using 10 µM as maximal oxysterol final concentration in the cell incubation medium. In the latter amount, 27OHC caused about a 20% fall in cell viability, after 48 h of treatment, while 5-µM 27OHC only slightly affected this parameter (Figure 2, Panel A). With regard to mitotane, after the 48-h cell challenge, a 5 µM final concentration reduced of about 10% cell viability as for 5-µM 27OHC, while a 10 µM concentration affected cell viability slightly more than 10-µM 27OHC (Figure 2, panel A).

### 3.3. Impairment of Mitochondrial Transmembrane Potential by Mitotane and 27OHC

Since mitotane was already known to impair mitochondrial function [7], the possible effect on mitochondrial transmembrane potential by the employed dose of this drug was then assessed. Indeed, a fairly moderate decrease in the aggregate fluorescent count, showing a depolarization in mitochondrial membrane, was observed after both 5- and 10-µM mitotane treatments, being statistically significant at the higher drug’s dosing (Figure 2, Panel B). An almost identical impairment of mitochondrial transmembrane potential was detectable when H295R cells were incubated for 48 h at 37 °C in the presence of either 5 µM and 10 µM 27OHC. In this case also, a statistical significance was achieved at the oxysterol’s higher concentration (Figure 2, panel B).

### 3.4. Apoptotic Effect of Mitotane and 27OHC on H295R Cells

The mitotane treatment carried out on the employed cancer cell line showed to be markedly effective in activating caspase-3 after 48 h, moderately increasing in 10-µM-treated cells in comparison to 5-µM-treated ones (Figure 2, Panel C). Notably, identical micromolar concentrations of 27OHC perfectly matched the mitotane induced caspase-3 activation, with no apparent difference in efficacy between the two concentrations used (Figure 2, Panel C).

### 3.5. Changes in Cholesterol Synthetic and Oxidative Pathways in ACC Patients

A number of relevant metabolic alterations were observed in the recruited patients undergoing mitotane treatment as compared to the disease-free individuals.

Despite no correlation between mitotane and cholesterol blood levels being observed in ACC patients (Table 1), the upstream cholesterol precursor lanosterol and the downstream cholesterol precursor desmosterol showed 2.8- and 2.6-fold significant increases in the plasma from mitotane-treated ACC patients compared to controls, respectively (Table 3). A much higher value was observed for lathosterol, the other key downstream cholesterol precursor, which resulted in four-fold increase compared with controls.

Moreover, plasma levels of the three oxysterols of enzymatic origin and of major pathophysiological interest exhibited quite a different behavior in mitotane treated neoplastic patients in comparison to control (Table 3). A modest increment, just statistically significant as compared to the control, was observed in neoplastic patients with regard to plasma 25OHC level. On the contrary, a much higher and more statistically meaningful increase in ACC group was shown by plasma 24OHC and by 27OHC. Notably, even though no correlation between 27OHC and cholesterol plasma levels was found in the single ACC patient, 24OHC doubled the control mean value, while the 27OHC plasma level reached an increase of 3.6 times.

## 4. Discussion

A detailed investigation of the molecular mechanisms that underlie the therapeutic effect of mitotane on ACC is necessary to improve the clinical handling of the drug. Along this line, the elucidation of mitotane modulation of cholesterol oxidation pathway appears useful. In a comprehensive in vitro study of the specific lipid changes provoked by mitotane, Sbiera and colleagues recently showed, for the first time, that this drug is able to perturbate the cholesterol oxidation pathway [13]. Namely, they reported that the challenge of H295 cells with a single dose of 50 or 100 µM mitotane significantly increased the intracellular steady-state level of 7KC and 7βOHC as to mitotane untreated cells [13]. Only these two oxysterols of non-enzymatic origin were measured, as the study was not focused on oxidative cholesterol metabolism.

This observation prompted us to investigate mitotane effect on the main oxysterols of recognized pathophysiological meaning, not only in vitro but also in vivo. The most meaningful finding both in in vitro (Table 2 and Figure 1) and in vivo (Table 3) was the fact that the treatment with mitotane led to a marked increase of 27-hydroxycholesterol, as measured by GC-MS standard methodology. The oxysterol 27OHC is the product of cholesterol oxidation by the ubiquitous and constitutive mitochondrial hydroxylase CYP27A1 [20] and represents the most abundant oxysterol in human plasma [21]. The role of this oxysterol in cancer is highly debated and related literature is quite controversial, given that different experimental approaches and conditions were employed, a wide variety of cancer types was assessed [22], and the oxysterol content varied significantly in the single tumors [23]. As far as human adrenal cancer cells are concerned, this paper provides solid evidence of specific cytotoxic and pro-apoptotic actions of 27OHC, which matches the in vitro effect exerted by an identical micromolar amount of mitotane (Figure 2). Indeed, a significant contribution of 27OHC to the anti-ACC action of mitotane could be viewed as very likely. The higher cytotoxicity of mitotane as to 27OHC observed in H295 cells treated with 10 µM concentration (Figure 2, Panel A), could be due to a derangement of plasma membrane ion channels, similar to that exerted by DDT, the mitotane parental compound. This hypothesis will be certainly checked in future studies.

The content of two other side-chain oxysterols—24OHC and 25OHC—increased, even if it was to a lesser extent, in ACC patients (Table 3). Their possible involvement in the therapeutic effect of mitotane cannot be excluded, even though in the standard in vitro ACC model, i.e., the H295R cell line, they were not detectable (Table 2).

Of note, as shown in Table 2, no significant increase of 7KC and 7βOHC in mitotane-treated as to untreated H295R cells was detected. Such a diverging finding from that reported by Sbiera and colleagues using the same cell line [13] could be, at least in part, due to the markedly different concentration range of mitotane adopted and its related cytotoxicity.

In the present paper, the cell treatment consisted of 5 or 10 µM mitotane, the latter affecting cell viability by 30% at 48 h incubation (Figure 2, Panel A), while in the quoted report mitotane concentrations ranged between 50 and 100 µM [13], i.e., one order of magnitude higher compared to those employed in the present experiments. At such relatively high drug concentrations, cell viability was reduced by 80% after 24 h incubation [13]. This cell condition could have favored the non-enzymatic oxidation of cholesterol with a consequent rise of 7KC and 7βOHC. Side chain oxysterols were not considered in that study.

With regard to the cholesterol synthesis pathway, a 48 h challenge of H295R cells with 10 µM mitotane induced a 1.5 times higher amount of cholesterol in comparison to untreated cells (see Results, Sub-Chapter 3.2), with a net increase in lanosterol, upstream cholesterol precursor, and in lathosterol (Table 2), the intermediate metabolite of the Kandutsch–Russell pathway of cholesterol synthesis. The parallel drop of desmosterol, the final intermediate of the Bloch pathway of cholesterol synthesis, confirmed that in the H295 cell line the first of the two pathways was, by far, prevalent. These in vitro findings, in this case, corresponded to those obtained by Sbiera and colleagues [13]. It is of interest that the quantification of cholesterol precursors in the plasma of our ACC patients demonstrated an increased activation of both cholesterol synthetic pathways during the treatment with mitotane (Table 3), while total cholesterol levels showed a wide variability in the different patients (Table 1). Moreover, no correlation was found in the individual patients between mitotane and cholesterol or 27OHC at least at the single time point considered in this study. Future studies will imply a larger number of patients and prospective follow-up evaluations.

Our samples included only patients with Stage I or II ACC, and this is explained by three reasons: (i) our institution is a referral center in Italy for ACC treatment with specific expertise in adjuvant mitotane therapy; therefore, our recruitment pattern consists of early stage ACC patients in more than half of the cases [24]; (ii) patients with advanced disease are often treated with the association of mitotane and chemotherapy [25], which could have interfered with our analysis; (iii) patients with Stage I and II have higher chances of survival [3], allowing a better appraisal of mitotane effect. It is known that administration of mitotane is aimed to target plasma mitotane concentrations between 14 and 20 mg/L, which have been associated with the drug efficacy (“therapeutic window”) [24,25,26,27], but during follow-up there are fluctuations of plasma mitotane levels. A prospective study is necessary to evaluate changes of the 27OHC plasma concentrations in relation to variations of plasma mitotane values, which could potentially pave the way to the use of 27OHC levels to monitor the mitotane therapeutic response. Therefore, regarding the in vivo analyses, limitations refer to the limited number of ACC patients available for the study.

Moreover, it may be of interest to evaluate the changes of 27OHC levels after mitotane exposition in other cell lines also, focusing on the endocrine glands other than adrenals (i.e., pituitary) that are affected by mitotane both in vitro [28,29] and in vivo [30,31].

To summarize, the main findings of this study are: (1) mitotane treatment induces a net increase in 27OHC in ACC cells and in ACC patients; (2) 27OHC is responsible for at least part of the cytotoxic and pro-apoptotic effects exerted by mitotane on ACC cells.

## 5. Conclusions

GC-MS quantification of the main cholesterol precursors and oxysterols in the standard ACC cell line as well as in the plasma of ACC patients undergoing mitotane treatment confirms a marked modulation of steroidogenesis, and identifies a remarkable feature of mitotane action in the rise of 27-hydroxycholesterol. Only a prospective study will permit the evaluation of a possible correlation between the increase in 27OHC and the therapeutic efficacy of mitotane. Nevertheless, 27OHC appears to be a promising candidate marker of mitotane action.

Moreover, the novel findings provided here could shed further light on the mitotane mechanism of action. Part of the specific cytotoxic effect of mitotane on ACC cells seems related to the increased intracellular amount of 27OHC induced by the drug. Pertinently, this oxysterol was demonstrated to impair mitochondrial function and induce apoptosis in ACC cells to a similar extent as mitotane.

## Figures and Tables

**Table 1:** Clinical features of adrenocortical carcinoma (ACC) patients.

Subjects	Gender	ACC Stage	Disease Status	Mitotane	Mitotane Levels (µg/mL)	Total Chol. Levels (mg/dL)	Cortisol Levels (µg/dL)
1	M	II	NED	yes	7.47	218	2.20
2	F	I	NED	yes	9.55	251	1.50
3	M	II	AWD-DOD	yes	9.97	197	22.30
4	F	II	AWD-DOD	yes	7.04	334	1.83
5	F	I	NED	yes	10.85	259	1.86
6	F	II	NED	yes	8.15	216	<1
7	F	I	NED	yes	4.12	261	12.80
8	F	II	NED	yes	13.29	251	6.75
9	F	II	NED	yes	19.36	225	2.37
10	F	II	NED	yes	14.19	246	1.20
11	F	II	NED	yes	15.94	204	2.35
12	M	II	NED	yes	14.43	202	2.56
13	F	II	AWD	yes	14.59	259	2.11
14	F	II	NED	yes	17.89	464	<1

M: male; F: female; I, II stages: see Methods 2.2; NED: No Evidence of Disease; AWD: Alive With Disease; DOD: Dead Of Disease.

**Table 2:** Cholesterol, cholesterol precursors and oxysterols in H295R cells.

Sterols	Untreated H295R Cells	Mitotane Treated H295R Cells
	µg/10^8^ cells	µg/10^8^ cells
Cholesterol	106 ± 30	157 ± 56 ***
	ng/10^8^ cells	ng/10^8^ cells
Lanosterol	1092 ± 44	1897 ± 176 ***
Desmosterol	4290 ± 244	865 ± 43 ***
Lathosterol	1040 ± 69	1631 ± 147 ***
7-Ketocholesterol	55 ± 1	50 ± 3
7α-hydroxycholesterol	34 ± 2	21 ± 8 *
7β-hydroxycholesterol	34 ± 1	25 ± 4
24-hydroxycholesterol	n.d.	n.d.
25-hydroxycholesterol	n.d.	n.d.
27-hydroxycholesterol	28 ± 5	130 ± 5 ***

H295R cells were treated with 10 µM mitotane for 48 h and GC-MS analyses were evaluated on collected cell pellets. Data are referred as means ± SD of three independent experiments; n.d.: not detectable. Significantly different versus untreated cells: * *p* < 0.05, *** *p* < 0.001.

**Table 3:** Profiling of key cholesterol precursors and of enzymatic oxysterols in plasma of controls and ACC patients.

Subjects	Lanosterol (ug/L)	Desmosterol (ug/L)	Lathosterol (ug/L)	24OHC (ug/L)	25OHC (ug/L)	27OHC (ug/L)
Controls (n = 5)	65 ± 7	522 ± 59	764 ± 40	22 ± 6	11 ± 5	39 ± 3
ACC post-mitotane (n = 14)	186 ± 83 **	1339 ± 539	3054 ± 872 ***	48 ± 13 ***	18 ± 5 *	142 ± 34 ***

Number of subjects are reported in parentheses (n). Plasma transfusion bags were used for control analyses. Values are means ± SD. Significantly different versus controls: * *p* < 0.05, ** *p* < 0.01, *** *p* < 0.001.

## Abbreviations

ACC: adrenocortical carcinoma; Ac-DEVD-AMC: N-Acetyl-Asp-Glu-Val-Asp-7-amino-4-methylcoumarin; AMC: 7-amino-4-methylcoumarin; ATCC: American Type Culture Collection; BSTFA: N,O-Bis(trimethylsilyl)trifluoroacetamide; 27OHC: 27-hydroxycholesterol; 7KC: 7-ketocholesterol; 7αOHC: 7α-hydroxycholesterol; 7βOHC: 7β-hydroxycholesterol; 24OHC: 24-hydroxycholesterol; 25OHC: 25-hydroxycholesterol; CYP450: cytochrome P450; DDT: dichlorodiphenyltrichloroethane; DMEM/F12: Dulbecco’s Modified Eagle’s Medium and Ham’s F-12 Nutrient mixture; EDTA: ethylenediaminetetraacetic acid; ER: endoplasmic reticulum; GC-MS: gas chromatography-mass spectrometry; HEPES: 4-2-hydroxyethyl-1-piperazineethansulphonic acid; JC-1: 5,5′,6,6′-tetrachloro1,1′,3,3′-tetraethylbenzimidazolylcarbocyanine iodide; PBS: phosphate buffered saline.
